# Editorial: The Role of TNF-TNFR2 Signal in Immunosuppressive Cells and Its Therapeutic Implications

**DOI:** 10.3389/fimmu.2019.02126

**Published:** 2019-09-06

**Authors:** Xin Chen, Magdalena Plebanski

**Affiliations:** ^1^State Key Laboratory of Quality Research in Chinese Medicine, Institute of Chinese Medical Sciences, University of Macau, Macau SAR, China; ^2^School of Health and Biomedical Sciences, RMIT University, Bundoora, VIC, Australia

**Keywords:** TNF, TNFR2, CD4^+^Foxp3^+^ regulatory T cells, myeloid-derived suppressive cells, mesenchymal stem cells, inflammation, cancer, autoimmune diseases

TNF is generally believed to be a master pro-inflammatory cytokine, and anti-TNF therapy has become the mainstream treatment for some autoimmune diseases. However, experimental evidence indicates that TNF preferentially activates Tregs, resulting in their proliferation, and enhancing their phenotypic stability and suppressive capacity. This effect of TNF is mediated by the cell surface receptor TNFR2, which is highly expressed by both human and mouse Tregs (1–4). Furthermore, within Tregs, expression of TNFR2 identifies the most suppressive fraction of cells (5). This Research Topic summarizes the latest knowledge on the critical role that TNF-TNFR2 signaling play in modulating the biology of Tregs, as well as other immune-suppressive cell types. Moreover, it analyzes its implications in controlling harmful inflammatory responses, as well as the potential targeting of this key signaling pathway to develop new immunotherapies against cancer.

## Mechanism of TNFR2 Signaling in the Activation and Expansion of Tregs

Although there is compelling evidence that TNF signaling through the TNFR2 receptor preferentially stimulates Tregs, the molecular mechanism had remained largely unknown. The study of human Tregs by Urbano et al. from Radboud University sheds some new light on this important topic. The results from their *in vitro* studies indicate that, through epigenetic regulatory pathways, the autocrine TNF-TNFR2 feedback loop promotes the stability of a highly suppressive phenotype for Tregs, namely one that expresses high levels of TIGIT, FOXP3, Helios, and EZH2. The study by He et al. from the University of Macau in turn provides both *in vitro* and *in vivo* experimental evidence that P38 MAPK is a component of the signaling pathway, which leads to TNFR2 mediated expansion of Tregs in response to TNF stimulation, as demonstrated by using small molecule inhibitors of known TNFR2 signaling pathways in T cells. The implications of the existence of such signaling pathways in disease are discussed by Yang et al. from Sun Yat-sen University, summarizing prior knowledge on the signaling pathways pertaining to TNF-TNFR1 and TNF-TNFR2 interactions in the context of autoimmune diseases and tissue regeneration. The main conclusion was that targeting TNFR1 or TNFR2 signaling pathways has significant therapeutic implications.

## Role of TNFR2-expressing Tregs in Cancer Immunology

The tumor microenvironment is often rich in both TNF, as well as in TNFR2-expressing immune cells, specifically immunosuppressive Tregs or myeloid-derived suppressor cells (MDSCs) (6, 7). High expression of TNFR2 is a characteristic of tumor associated Tregs that potently inhibit anti-tumor immune responses in many different types of cancer (5, 8–10). This idea is further substantiated by a study on patients with advanced epithelial ovarian cancer, by Kampan et al. from Monash University. Furthermore, this study found that IL-6 present in malignant ovarian cancer ascites is responsible for the up-regulation of TNFR2 expression and the expansion of highly suppressive Treg subsets. This finding may help to devise novel immunotherapies aiming to eliminate indirectly tumor-associated immune-suppressive Treg activity by inhibiting IL-6. Salomon et al. from Centre d'Immunologie et des Maladies Infectieuses discussed both historic and current understanding of the puzzling role of TNF as well as anti-TNF therapy in immune and inflammatory responses, leading up to our current understanding of the TNF-TNFR2 pathway and its decisive role in controlling the activation of Tregs. Through the analysis of recent reports on the therapeutic use of agonistic TNFR2-targeting agents in graft vs. host disease (GvHD), this review article raises the possibility of targeting of TNFR2-expressing Tregs using such agents, as potentially a safe and efficient approach to enhance anti-tumor immunity. In addition to its expression on immunosuppressive Tregs, TNFR2 can also be expressed by some tumor cells. Sheng et al. from Zhengzhou University summarized recent research regarding the role of TNFR2 in the promotion of carcinogenesis, cancer immune evasion and tumor growth. They concluded that TNFR2 was an ideal candidate for targeted tumor therapy. Moreover, this article clearly explains bi-directional signaling via TNFR2, given membrane-bound TNF (mTNF) preferentially binds to and activates TNFR2, and in addition to the forward signaling of mTNF → TNFR2, reverse signaling (e.g., TNFR2 → mTNF) can also occur. In this case, mTNF acts as a receptor that can transduce activating intracellular signaling when interacting with either sTNFR2 or membrane-bound TNFR2 on the surface of cells. Qu et al. from Tianjin Medical University further takes up this discussion into the possible role of forward and reverse crosstalk between mTNF and TNFR2 in an immunosuppressive tumor microenvironment. Indeed, these reviews leave open the question of whether reverse signaling by TNFR2 expressing Tregs may substantially affect mTNF-expressing immune cells and tumor cells.

## The Role of TNFR2-expressing Tregs in Other Diseases

The significant role that TNFR2-expressing Tregs can play in the pathogenesis and treatment of other types of diseases are also included in this Research Topic. Pegoretti et al. from University of Groningen discussed the possibility of selectively modulating the individual TNF receptors, TNFR2 or TNFR1, for the treatment of multiple sclerosis (MS), a neurodegenerative autoimmune disease which is currently resistant to anti-TNF treatment. Mancusi et al. from the University of Perugia discussed the need to evaluate whether Treg activation via TNFR2 could be used to practically enhance the yield, purity, and/or efficacy of Tregs used for cell therapy. Furthermore, they propose that such an approach has the potential for quick clinical translation in HSCT trials, since it is reported that Tregs have the capacity to prevent GvHD and promote immune reconstitution. Ahmad et al. from Universiti Sains Malaysia analyzed the role of TNF-TNFR2 interactions in immune tolerance to allergens and concluded that targeting TNF-TNFR2 interactions may represent a novel strategy for the treatment of allergic inflammatory responses. Based on the idea that genetic variation in the promoter of the TNFRSF1B gene could have a major impact on disease susceptibility, as well as potential responsiveness to TNFR2-targeting therapies, Li and Anderson from National Cancer Institute at Frederick proposed a key transcription factor binding site that may have significant effects on TNFRSF1B promoter activity, and suggested that it should be considered in future studies.

## The Role TNFR2 Signaling in Other Type of Immunosuppressive Cells

In addition to conventional CD4^+^ Tregs, TNFR2 also plays a key role in modulating the activity of other type of immunosuppressive cells, such as myeloid-derived suppressive cells (MDSCs), Mesenchymal stem cells (MSCs) and CD8^+^ Treg cells. In this Research Topic, Chavez-Galan et al. from University of Geneva studied the role that mTNF may play a role in promoting accumulation and enhancing function of MDSC in the pleural cavity during an acute mycobacterial infection. They found that the interaction of mTNF expressed by MDSCs and TNFR2 expressed by CD4 T cells is required for protection against the lethal inflammatory responses, which are sometimes associated with pleural mycobacterial infection. However, Schmid et al. from the University of Regensburg did not observe significant effects on MDSCs after TNFR2 agonist treatment *in vivo*. In addition to CD4 Tregs, some TNFR2-expressing CD8 T cells also have suppressive capacity. Ye et al. from Huazhong University of Science and Technology analyzed the functional consequences of TNFR2 expression by CD8^+^Foxp3^+^ Tregs and by CD8^+^ Teffs, and concluded that a TNF-TNFR2 mediated coordinated complex events ultimately result in strong CD8^+^ T cell-mediated immune responses. Mesenchymal stem cells (MSCs) have immunosuppressive properties which may be therapeutically harnessed in the treatment of autoimmune diseases. Yan et al. from the University of Macau discusses the role of TNF signaling through TNFR1 and TNFR2 on the biology of MSCs. The effect of TNF in MSC-based therapy for autoimmune and inflammatory diseases is also discussed.

## Effect of TNFR2-targeting Pharmacological Agents on Treg Activity

Synthetic glycine coated 50 nm polystyrene nanoparticles (PS50G) are able to inhibit allergic airway inflammation. Mohamud et al. from Monash University reported that PS50G treatment preferentially stimulates the expansion of highly suppressive TNFR2^+^ Tregs, which express high levels of Ki-67, LAP, and CTLA-4. This is likely caused by the induction of CD103^+^ DCs in mice treated with PS50G. This property of engineered nanoparticles may prove to be useful in the treatment of inflammatory human diseases. Urbano et al. from Radboud University reported that the combination treatment with rapamycin and a TNFR2 agonist antibody was able enhance hypo-methylation of the FOXP3 gene, and consequently promote the stability of Tregs. Clear therapeutic potential has spurred the development of agonistic or antagonistic TNFR2-targeting biological agents in the recent years. Zou et al. from the University of Macau provides an overview regarding the latest progress in the study of TNFR2-targeting pharmacological agents and their therapeutic potential through the up- or down-regulation of Treg activity. In addition to protein drugs, this review suggests that small molecule inhibitors of TNFR2 may also have therapeutic value. Shaikh et al. from University of Macau performed a virtual screening of 400,000 naturally occurring small molecule compounds against TNF-binding sites of TNFR2. Their results indicate that a number of compounds could block the ligand-binding site of TNFR2. *In vitro* and *in vivo* studies are now needed to verify the results of this virtual study. Progranulin (PGRN) is a protein with immunosuppressive properties, which purportedly inhibits TNF-induced TNFR1/2 signaling by directly binding to TNFR1 and TNFR2. However, Lang et al. from University Hospital of Würzburg didn't observe a direct interaction between PGRN and TNFR1/2 in cellular binding studies.

Taken together, 19 primary research reports and review articles in this Frontiers Topic further support and substantiate the decisive role of TNF-TNFR2 interactions in promoting the activation, expansion and phenotypic stability of Tregs, as well as other immunosuppressive cells which may further include CD8 Tregs, MDSCs and MSCs. The critical role of TNFR2 signaling in helping maintain immune homeostasis, in promoting an immunosuppressive tumor microenvironment and in dampening autoimmune or allergic responses, is highlighted across these articles, as is an exploration of the molecular mechanisms that underlie this interaction. Emerging trends in the development of TNFR2-targeting therapeutics are further highlighted. We thus believe this article collection will be helpful for investigators performing fundamental research, as well clinical researchers. Given the substantial potential that targeting TNF-TNFR2 signaling offers for treatment of multiple diseases, we hope this collection of articles will spur further research, and eventually lead to new useful treatments.

## Author Contributions

All authors listed have made a substantial, direct and intellectual contribution to the work, and approved it for publication.

### Conflict of Interest Statement

The authors declare that the research was conducted in the absence of any commercial or financial relationships that could be construed as a potential conflict of interest.
